# Development and evaluation of a nomogram model for predicting malnutrition in patients with colorectal cancer

**DOI:** 10.3389/fmed.2025.1637579

**Published:** 2025-10-29

**Authors:** Ran Xu, Li-Fang Gong

**Affiliations:** ^1^College of Nursing, Chengdu University of Traditional Chinese Medicine, Chengdu, Sichuan, China; ^2^Emergency and Critical Care Center, Intensive Care Unit, Department of Nursing, Zhejiang Provincial People’s Hospital (Affiliated People’s Hospital), Hangzhou Medical College, Hangzhou, Zhejiang, China

**Keywords:** colorectal cancer, malnutrition, nomogram, GLIM criteria, predictive model, logistic regression

## Abstract

**Background:**

Malnutrition is a common complication in patients with colorectal cancer (CRC), negatively impacting treatment outcomes and quality of life. Early identification of patients at risk of malnutrition can aid in timely interventions. The objective of this study was to develop and evaluate a nomogram model for predicting malnutrition in CRC patients.

**Methods:**

This retrospective study was conducted at our hospital from January 2022 to December 2024. Nutritional assessments were based on parameters such as body mass index (BMI), serum albumin (ALB), hemoglobin (HGB), prognostic nutritional index (PNI), and others. Univariate logistic regression analysis was initially performed to identify potential risk factors for malnutrition. Statistically significant factors (*p* < 0.05) were included in a multivariate logistic regression model, which was used to construct a nomogram for predicting malnutrition risk. The nomogram’s performance was evaluated using the area under the curve (AUC) from receiver operating characteristic (ROC) analysis, calibration curves, and decision curve analysis (DCA).

**Results:**

Multivariate analysis identified six independent predictors: age ≥65 years (OR = 2.216, 95% CI: 1.312–3.843, *p* = 0.003), TNM stage IV (OR = 1.886, 95% CI: 1.091–3.278, *p* = 0.025), Karnofsky Performance Status (KPS) ≤80 (OR = 2.581, 95% CI: 1.525–4.368, *p* < 0.001), hemoglobin <110 g/L (OR = 0.317, 95% CI: 0.185–0.561, p < 0.001), prealbumin <200 g/L (OR = 0.513, 95% CI: 0.281–0.902, *p* = 0.020), and prolonged bed rest (OR = 9.739, 95% CI: 2.834–31.187, *p* < 0.001). The nomogram demonstrated good discrimination with an area under the curve (AUC) of 0.819 (95% CI: 0.731–0.895), sensitivity of 71.3%, specificity of 86.6%, and negative predictive value of 89.6%. Calibration was excellent (Hosmer–Lemeshow *p* = 0.929; *C*-index = 0.798). Decision curve analysis confirmed favorable clinical utility.

**Conclusion:**

The nomogram model, incorporating six risk factors, offers a reliable and effective tool for predicting malnutrition in CRC patients. It provides clinicians with an important decision-making aid for early intervention and management of malnutrition.

## 1 Introduction

Colorectal cancer (CRC) remains a significant global public health issue due to its high incidence and substantial morbidity. Although advancements in screening programs and therapeutic strategies have improved survival outcomes, malnutrition continues to be a common and frequently under-recognized complication that negatively impacts clinical prognosis in this population (1). In CRC patients, malnutrition may result from various factors, including reduced dietary intake, treatment-related toxicities, and metabolic disturbances associated with tumor-induced inflammation. Evidence indicates that malnourished patients experience more frequent treatment interruptions, extended hospital stays, poorer tolerance to surgical and chemotherapeutic interventions, and a reduced quality of life (2, 3). Therefore, accurate assessment and early prediction of malnutrition risk in CRC patients are essential for timely nutritional intervention and optimal clinical management. Malnutrition in CRC can arise from multiple factors, including reduced food intake, treatment-induced toxicities, and metabolic imbalances driven by tumor-induced inflammation (4). Patients with malnutrition have been shown to experience more frequent treatment interruptions, prolonged hospital stays, poorer tolerance to surgical and chemotherapeutic interventions, and diminished quality of life (5).

Several tools, including the Nutritional Risk Screening 2002 (NRS-2002), the Patient-Generated Subjective Global Assessment (PG-SGA), and the Global Leadership Initiative on Malnutrition (GLIM) criteria, have been widely utilized to assess nutritional status in clinical practice (6–8). These methods incorporate a range of anthropometric, biochemical, and functional parameters; however, they may fall short in integrating multiple risk factors into a unified, individualized predictive framework (9, 10). Traditional screening instruments typically provide categorical classifications of nutritional risk, which can lead to oversimplified assessments and may fail to capture the complexity of disease progression or patient-specific clinical conditions (11, 12). Moreover, prior studies have indicated that the sensitivity and specificity of these screening tools vary across clinical settings and patient populations, highlighting the need for prediction models tailored to specific cancer cohorts. Recent research has demonstrated the utility of nomograms in oncology for estimating individualized risk and clinical outcomes. A nomogram is a graphical representation of a multivariable predictive model that translates statistical results into a clinically applicable tool. By incorporating diverse variables, nomograms provide patient-specific estimates of clinical events and can be adapted to different disease contexts. In CRC, a nomogram model may integrate demographic characteristics, tumor stage, comorbidities, and laboratory biomarkers to enhance the precision of malnutrition risk assessment (13, 14). This integrative approach has the potential to support timely, personalized nutritional interventions and reduce complications associated with malnutrition.

Against this backdrop, the present study aimed to develop and validate a novel nomogram model for predicting malnutrition in patients with CRC. By constructing and assessing this model, we sought to provide a practical and individualized clinical tool for identifying CRC patients at increased risk of malnutrition. The findings of this study may inform the development of targeted interventions and clinical protocols aimed at improving nutritional management, enhancing treatment tolerability, and optimizing overall patient outcomes.

## 2 Methods

### 2.1 Study design

This retrospective study was conducted at our hospital from January 2022 to December 2024 to develop and evaluate a nomogram model for predicting malnutrition in patients with colorectal cancer. Patients included in the study met the following criteria: (1) a confirmed pathological diagnosis of colorectal cancer based on histological examination; (2) aged 18 years or older; and (3) a Karnofsky Performance Status (KPS) score of ≥60. Additionally, participants were required to have comprehensive and accurate nutritional assessments available, including body mass index (BMI), serum albumin levels, and Nutritional Risk Screening scores. Exclusion criteria were as follows: (1) a history of secondary malignancies or coexisting cancers unrelated to colorectal cancer; (2) severe comorbidities, including significant cardiovascular, hepatic, renal, or respiratory conditions that could independently affect nutritional status; and (3) incomplete or missing clinical or nutritional data essential for the development of the model. A total of 216 patients with colorectal cancer were included in the study, comprising 56 patients classified as malnourished and 160 patients classified as well-nourished based on established criteria. The study protocol adhered to the STROBE (Strengthening the Reporting of Observational Studies in Epidemiology) guidelines (15). Informed consent was obtained from all participants or their legal guardians. The study was reviewed and approved by the hospital’s ethics committee (2025-0107-009), and conducted in accordance with relevant guidelines and the Declaration of Helsinki. All data was kept confidential, with personal identifiers removed prior to analysis to ensure participant privacy.

### 2.2 Diagnostic criteria for malnutrition using the GLIM framework

This study adopted the GLIM criteria for the diagnosis of malnutrition (16). The diagnostic process involved a two-step approach: (1) Screening for malnutrition risk: The NRS-2002 tool was utilized as the primary screening instrument. Patients with an NRS-2002 score of ≥3 were identified as being at risk for malnutrition through a review of medical records. (2) Assessment of malnutrition: Patients identified as at risk in the screening step underwent a detailed malnutrition assessment based on the GLIM criteria. The assessment comprised five components grouped into two categories, with patients being classified as malnourished if they fulfilled at least one phenotypic criterion and one etiologic criterion.

Phenotypic criteria: (1) Non-volitional weight loss: A weight loss of >5% within six months or >10% beyond six months. (2) Low body mass index (BMI): Defined according to Asian-specific thresholds—BMI <18.5 kg/m^2^ for individuals under 70 years of age and BMI <20 kg/m^2^ for those aged 70 years or older (17). (3) Reduced muscle mass: Identified as below normal values measured by body composition analysis techniques.

Etiologic criteria: (1) Reduced food intake or absorption: Food intake <50% of normal for over one-week, reduced intake for more than 2 weeks, or chronic gastrointestinal dysfunction impairing digestion or absorption. (2) Disease burden or inflammation: Acute disease- or trauma-related inflammation, or chronic disease-associated inflammation.

### 2.3 Data collection from hospital and laboratory information systems

Comprehensive patient data were collected using the Hospital Information System and Laboratory Information Management System. The dataset included demographic, clinical, and biochemical parameters to ensure a robust analysis. The collected variables were as follows:

1) Demographic and lifestyle information: Gender, residence, marital status, and history of prolonged bedridden status.
2) Clinical characteristics: Age, Karnofsky Performance Status (KPS) score (18), tumor staging, body mass index (BMI), history of alcohol consumption, and gastrointestinal surgery within the past 2 years.
3) Comorbidities: Presence of hypertension, diabetes, and coronary artery disease.
4) Nutritional risk: Nutritional Risk Screening 2002 (NRS-2002) scores (19).
5) Hematological and biochemical parameters: (1) Hematological markers: Red blood cell count (RBC), white blood cell count (WBC), hemoglobin (HGB), and platelet count (PLT). (2) Nutritional markers: Serum albumin (ALB) and prealbumin (PAB). (3) Liver function indicators: Alanine aminotransferase (ALT) and aspartate aminotransferase (AST). (4) Renal function markers: Blood urea (Urea) and serum creatinine (Cr).

### 2.4 Statistical analysis

All statistical analyses were performed using SPSS version 27.0. Continuous variables with a normal distribution were expressed as mean ± standard deviation, and comparisons between groups were conducted using the independent t-test. Non-normally distributed data were presented as median (interquartile range) [M(Q₁, Q₃)], with group comparisons performed using the rank-sum test. Categorical variables were summarized as frequencies and percentages [*n* (%)], and differences between groups were analyzed using the chi-square (*χ*^2^) test. Univariate logistic regression analysis was initially conducted to identify potential risk factors with statistical significance. Variables with significant differences were then included in a multivariate logistic regression model. A nomogram was constructed using the R software package based on the significant predictors identified. The predictive performance of the model was evaluated by calculating the area under the receiver operating characteristic (ROC) curve (AUC). The discriminative ability of the model was categorized as follows: AUC values of 0.5–0.7 indicated low discrimination, >0.7–0.9 moderate discrimination, and >0.9–1.0 high discrimination. The concordance index (*C*-index) was also calculated as a measure of model discrimination, with values closer to 1.0 reflecting better predictive accuracy. The optimal cutoff value for the nomogram was determined using the Youden index, which maximizes the sum of sensitivity and specificity. Model calibration was assessed using the Hosmer–Lemeshow goodness-of-fit test, where a non-significant result (*p* > 0.05) indicates good agreement between predicted and observed outcomes. For interpretation of the nomogram, each predictor variable corresponds to a point score on the top scale. The individual points assigned to each variable are summed to obtain a total score, which is then projected onto the probability scale to estimate the individualized risk of malnutrition. Calibration curves were plotted to assess the concordance between predicted probabilities and observed outcomes. Clinical utility and applicability of the model were further evaluated using decision curve analysis (DCA). A *p*-value <0.05 was considered statistically significant.

## 3 Results

### 3.1 Baseline characteristics of malnourished and well-nourished patients

The baseline characteristics of patients in the malnourished and well-nourished groups are summarized in Table 1. Significant differences were observed between the two groups in several variables. A higher proportion of patients aged 65 years or older was found in the malnourished group (67.9%) compared to the well-nourished group (49.4%) (*χ*^2^ = 5.707, *p* = 0.017). In terms of tumor stage, a significantly higher percentage of patients in the malnourished group were at stage IV (73.2%) compared to the well-nourished group (51.2%) (*χ*^2^ = 8.162, *p* = 0.004). Additionally, prolonged bed rest, KPS scores, BMI, and NRS-2002 scores all showed significant differences between the two groups. The malnourished group had a higher incidence of prolonged bed rest, lower KPS scores, and lower BMI (all *p* < 0.05). The malnourished group also had significantly higher NRS-2002 scores, indicating greater nutritional risk. No significant differences were found in sex, marital status, drinking history, or recent gastrointestinal surgery.

**Table 1:** Baseline characteristics of patients in the malnourished and well-nourished groups.

General information	Category	Malnourished group (*n* = 56)	Well-nourished group (*n* = 160)	*χ* ^2^	*p*-value
Age	<65 years	18 (32.1)	81 (50.6)	5.707	0.017
	≥65 years	38 (67.9)	79 (49.4)		
Sex	Male	34 (60.7)	99 (61.9)	0.024	0.878
	Female	22 (39.3)	61 (38.1)		
Tumor stage	I–III	15 (26.8)	78 (48.8)	8.162	0.004
	IV	41 (73.2)	82 (51.2)		
Marital status	Married	45 (80.4)	138 (86.3)	1.113	0.292
	Other	11 (19.6)	22 (13.7)		
Drinking history	No	47 (83.9)	133 (83.1)	0.019	0.890
	Yes	9 (16.1)	27 (16.9)		
GI surgery (past 2 years)	No	45 (80.4)	131 (81.9)	0.063	0.801
	Yes	11 (19.6)	29 (18.1)		
Hypertension	No	39 (69.6)	105 (65.6)	0.301	0.583
	Yes	17 (30.4)	55 (34.4)		
Diabetes	No	49 (87.5)	135 (84.4)	0.321	0.571
	Yes	7 (12.5)	25 (15.6)		
Prolonged bed rest	No	10 (17.9)	2 (1.3)	21.80	<0.001
	Yes	46 (82.1)	158 (98.7)		
KPS	≥90	22 (39.3)	96 (60.0)	7.181	0.007
	≤80	34 (60.7)	64 (40.0)		
BMI	<18.5 kg/m^2^	4 (7.1)	2 (1.3)	11.66	0.003
	18.5–24.0 kg/m^2^	40 (71.4)	118 (73.8)		
	>24.0 kg/m^2^	12 (21.4)	40 (25.0)		
NRS-2002	0–2	20 (35.7)	138 (86.3)	53.94	<0.001
	≥3	36 (64.3)	22 (13.7)		

KPS, Karnofsky Performance Status; BMI, body mass index; GI, gastrointestinal; NRS-2002, Nutritional Risk Screening 2002.

### 3.2 Laboratory parameters comparison between malnourished and well-nourished groups

There were no significant differences in Cr and AST levels between the malnourished and well-nourished groups (*p* = 0.568 and *p* = 0.425, respectively). However, the malnourished group exhibited significantly lower ALT levels compared to the well-nourished group (*p* = 0.041). BUN levels were significantly higher in the malnourished group (*p* < 0.001). Regarding blood cell counts, the malnourished group had a similar PLT count compared to the well-nourished group (*p* = 0.112). However, the malnourished group showed significantly lower RBC count and HGB levels compared to the well-nourished group (*p* < 0.001 for both). Furthermore, ALB levels were significantly lower in the malnourished group (*p* < 0.001) (Table 2).

**Table 2:** Comparison of laboratory parameters between malnourished and well-nourished groups.

Index	Malnourished group (*n* = 56)	Well-nourished group (*n* = 160)	*t*/*Z*	*p*
Cr	68.80 (56.50, 83.80)	68.30 (59.10, 79.50)	0.557	0.568
AST	25.10 (16.80, 45.50)	26.20 (19.80, 38.50)	0.792	0.425
PLT	194.50 (145.00, 287.00)	192.00 (143.00, 255.00)	1.623	0.112
ALT	18.80 (12.20, 34.00)	22.30 (15.30, 33.80)	2.088	0.041
BUN	5.75 (4.30, 9.20)	5.20 (4.10, 6.70)	3.227	<0.001
WBC	6.80 (4.60, 9.70)	5.40 (4.00, 7.50)	3.415	<0.001
PAB	126.50 ± 54.10	171.30 ± 55.80	5.211	<0.001
RBC	3.65 (2.95, 4.20)	4.33 (3.75, 4.80)	6.358	<0.001
HGB	110.10 ± 27.90	130.90 ± 24.00	5.346	<0.001
ALB	31.80 ± 5.95	38.00 ± 5.10	7.490	<0.001

Cr, creatinine; AST, aspartate aminotransferase; PLT, platelet count; ALT, alanine aminotransferase; BUN, blood urea nitrogen; WBC, white blood cell; PAB, prealbumin; RBC, red blood cell; HGB, hemoglobin; ALB, albumin.

Values are presented as mean ± SD for normally distributed data and as median (Q1–Q3) for non-normally distributed data. Group comparisons were performed using independent *t*-test or Mann–Whitney *U* test (*Z* value), as appropriate.

### 3.3 Univariate logistic regression analysis of factors associated with malnutrition

The univariate logistic regression analysis revealed several factors significantly associated with malnutrition. TNM stage was found to be a significant predictor, with an OR of 2.645 (*p* < 0.001). Bed rest also showed a strong association, with an OR of 17.028 (*p* < 0.001). BUN levels were positively correlated with malnutrition, exhibiting an OR of 6.83 (*p* < 0.001). Additionally, ALT levels, age, and KPS score were significantly related to malnutrition, with OR values of 2.392 (*p* = 0.013), 2.297 (*p* = 0.001), and 2.22 (*p* = 0.001), respectively. WBC levels were also significantly associated with malnutrition (OR = 2.10, *p* = 0.008) (Table 3).

**Table 3:** Univariate logistic regression analysis of factors associated with malnutrition.

Factors	*β* value	Standard error value	OR value	95% CI for OR	*p*-value
TNM stage	0.972	0.252	2.645	1.635–4.288	<0.001
Bed rest	2.882	0.541	17.028	5.768–51.204	<0.001
BUN	1.921	0.362	6.83	3.302–14.107	<0.001
ALT	0.872	0.36	2.392	1.198–4.777	0.013
Age	0.832	0.229	2.297	1.452–3.624	0.001
KPS score	0.798	0.22	2.22	1.442–3.454	0.001
WBC	0.742	0.218	2.1	1.388–3.176	<0.001
PAB	−1.306	0.235	0.271	0.170–0.435	<0.001
RBC	−1.365	0.245	0.255	0.158–0.402	<0.001
HGB	−1.492	0.247	0.225	0.142–0.365	<0.001
ALB	−1.602	0.247	0.201	0.125–0.325	<0.001
BMI	−5.134	0.635	0.006	0.002–0.019	<0.001

TNM, tumor-node-metastasis; BUN, blood urea nitrogen; ALT, alanine aminotransferase; KPS, Karnofsky Performance Status; WBC, white blood cell; PAB, prealbumin; RBC, red blood cell; HGB, hemoglobin; ALB, albumin; BMI, body mass index; *β*, regression coefficient; OR, odds ratio; CI, confidence interval.

### 3.4 Multivariate logistic regression analysis of risk factors for malnutrition

Multivariate logistic regression analysis was conducted using the statistically significant variables identified in the univariate analysis as independent predictors. The results indicated that advanced tumor stage (TNM stage IV) was a significant risk factor for malnutrition, with patients in this stage having a higher likelihood of malnutrition compared to those in stages I-III (*p* = 0.025). Additionally, advanced age (≥65 years) was found to significantly contribute to malnutrition risk (*p* = 0.003). Other identified risk factors included a lower KPS score, prolonged bed rest, lower hemoglobin levels, and decreased serum PAB levels. All these factors were found to be independently associated with an increased risk of malnutrition (*p* < 0.05) (Table 4).

**Table 4:** Multivariate logistic regression analysis of factors associated with malnutrition.

Factors	Category	*β* value	Standard error value	OR value	95% CI for OR	*p*-value
TNM stage	I–III (reference)	—	—	—	—	—
	IV	0.634	0.288	1.886	1.091–3.278	0.025
Age	<65 years (reference)	—	—	—	—	—
	≥65 years	0.796	0.268	2.216	1.312–3.843	0.003
Bed rest	No (reference)	—	—	—	—	—
	Yes	2.276	0.624	9.739	2.834–31.187	<0.001
KPS score	>80 points (reference)	—	—	—	—	—
	≤80 points	0.948	0.26	2.581	1.525–4.368	<0.001
PAB	<200 g/L (reference)	—	—	—	—	—
	≥200 g/L	−0.668	0.297	0.513	0.281–0.902	0.02
HGB	<110 g/L (reference)	—	—	—	—	—
	≥110 g/L	−1.149	0.287	0.317	0.185–0.561	<0.001

TNM, tumor-node-metastasis; Age, chronological age; KPS, Karnofsky Performance Status; PAB, prealbumin; HGB, hemoglobin; *β*, regression coefficient; OR, odds ratio; CI, confidence interval.

### 3.5 Development of a nomogram for predicting malnutrition in CRC patients

**Figure 1:**
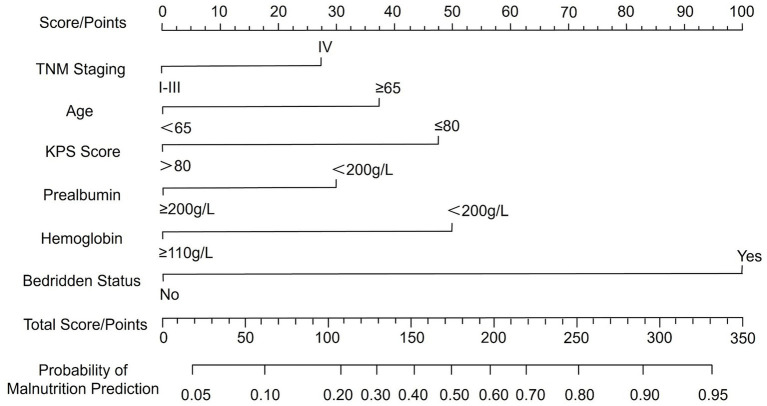
Nomogram for predicting malnutrition in patients with colorectal cancer.

A nomogram for predicting malnutrition in CRC patients was constructed based on the six significant factors identified through multivariate logistic regression analysis. These factors included TNM stage, age, KPS score, bed rest, hemoglobin levels, and serum PAB levels. For each variable, a corresponding score was assigned, and the cumulative score was then used to estimate the probability of malnutrition occurrence in individual patients. The total score, which was determined by summing the individual variable scores, directly corresponds to the likelihood of malnutrition, as reflected on the probability scale at the bottom of the nomogram. Higher total scores indicate a greater risk of malnutrition, thus allowing for more accurate risk stratification and early intervention in clinical practice to improve patient care and outcomes (Figure 1).

### 3.6 Discrimination of the nomogram prediction model

**Figure 2:**
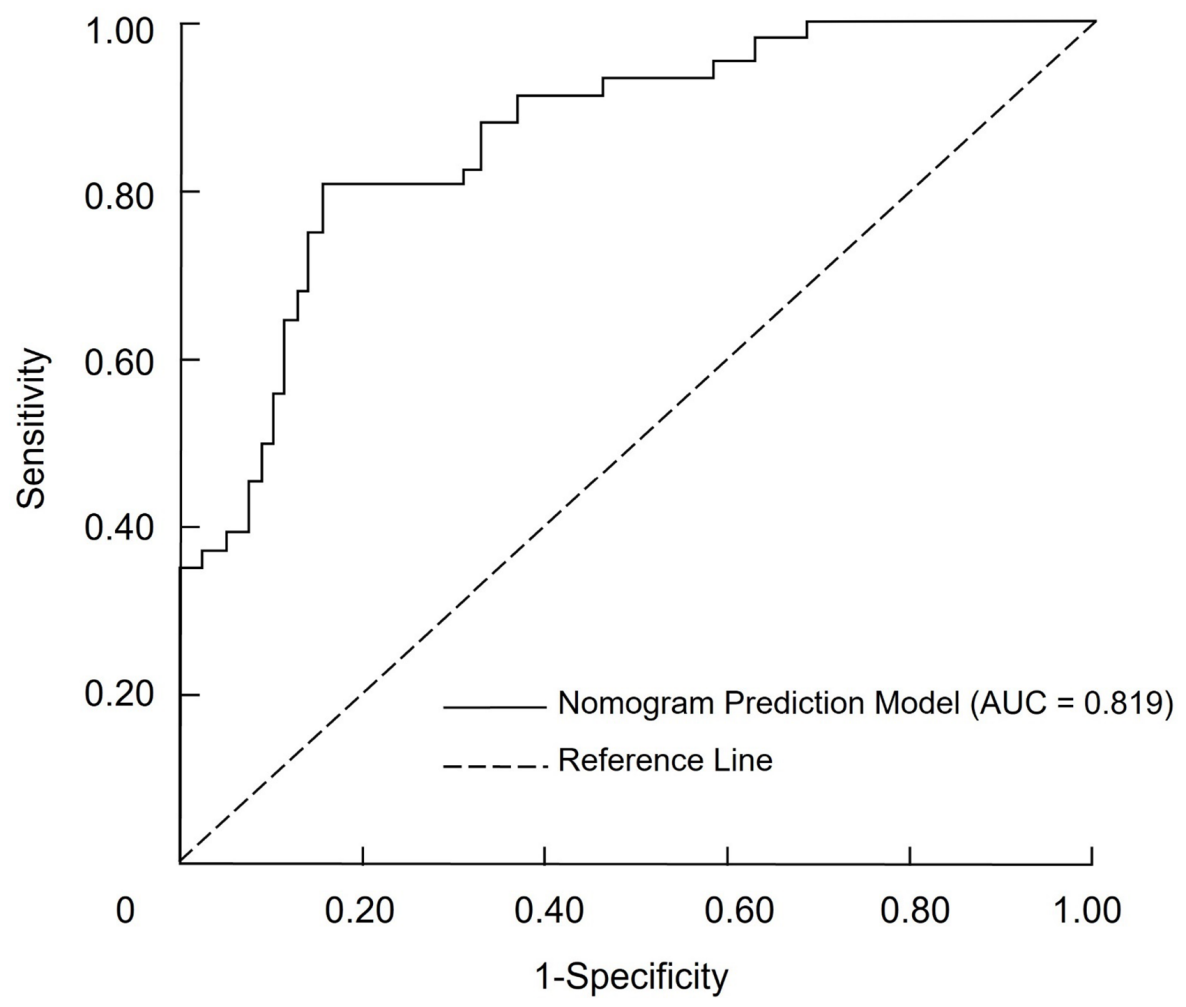
ROC curve for the nomogram model predicting malnutrition risk in CRC patients.

The discriminatory ability of the nomogram prediction model for malnutrition risk in patients with CRC was evaluated using the total risk score as the predictor variable and malnutrition occurrence as the outcome variable (Table 5). The model yielded an AUC of 0.819 (95% CI: 0.731–0.895), which indicates moderate discriminative ability according to conventional criteria. At the cutoff point defined by the maximum Youden index, the nomogram achieved a sensitivity of 71.29% and a specificity of 86.58%. The corresponding positive predictive value was 64.9%, and the negative predictive value was 89.6%, as illustrated in Figure 2.

**Table 5:** Diagnostic performance of the nomogram prediction model for malnutrition risk in patients with colorectal cancer.

Metric	Value
AUC	0.819 (95% CI: 0.731–0.895)
Sensitivity	71.3%
Specificity	86.6%
PPV	64.9%
NPV	89.6%

ROC, receiver operating characteristic; AUC, area under the curve; PPV, positive predictive value; NPV, negative predictive value; CRC, colorectal cancer.

### 3.7 Calibration of the nomogram prediction model

**Figure 3:**
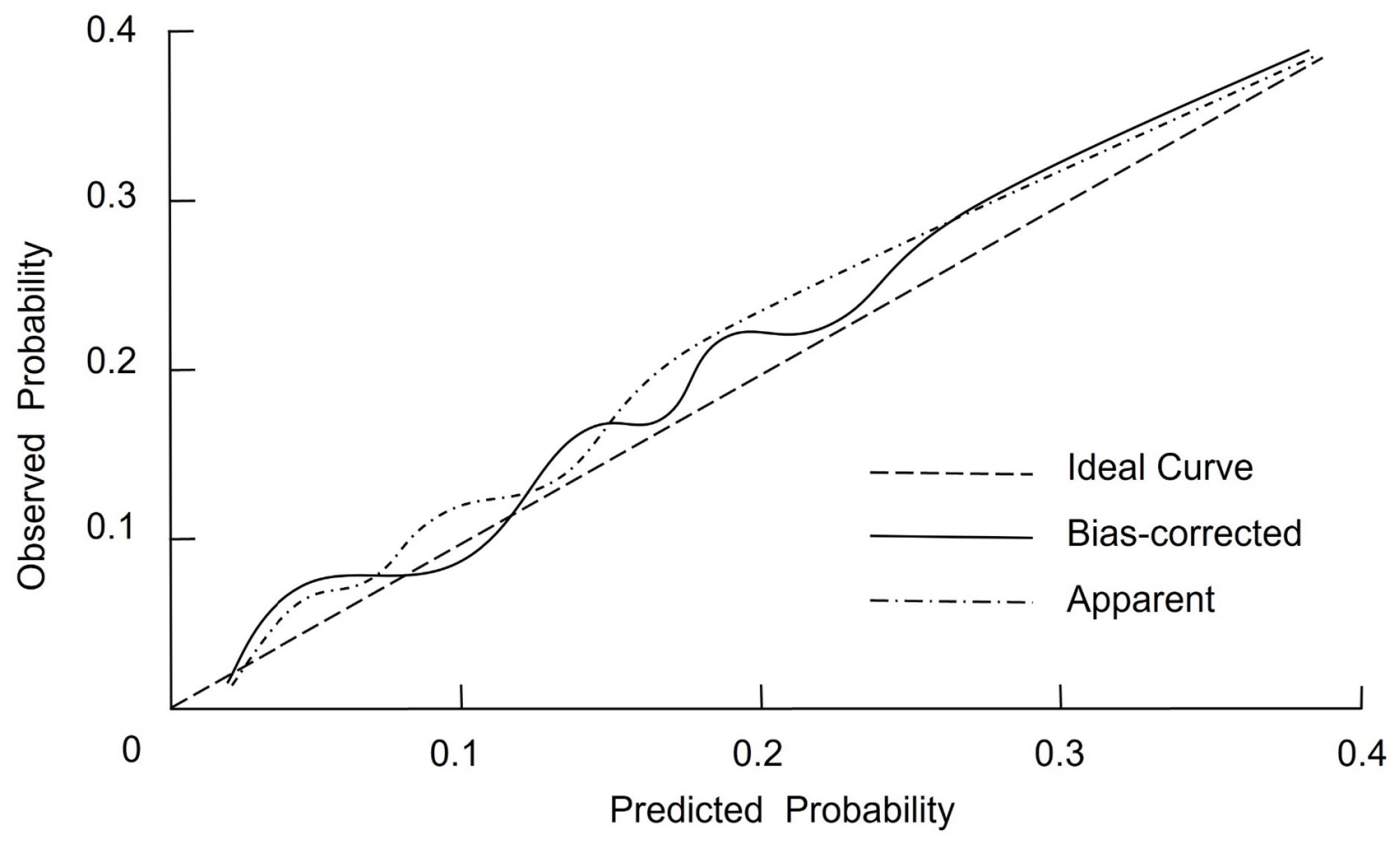
Calibration plot of the nomogram model for predicting malnutrition in CRC patients.

The internal validation of the nomogram prediction model was conducted using the Bootstrap resampling method with 1,000 iterations to reduce potential overfitting and assess model stability. The calibrated C-index was 0.798 (95% CI: 0.715–0.856), suggesting that the model possesses acceptable discriminative capacity in distinguishing between malnourished and well-nourished patients. Model calibration was further evaluated using the Hosmer–Lemeshow goodness-of-fit test, which yielded a *χ*^2^ value of 2.387 and a *p*-value of 0.929, indicating no significant deviation between predicted and observed outcomes. In addition, the calibration curve (Figure 3) showed that the bias-corrected predictions were closely aligned with the ideal reference line, while the apparent curve also demonstrated similar trends, confirming that the predicted probabilities were consistent with actual observations. Together, these findings support the reliability and robustness of the model in estimating individual malnutrition risk in CRC patients.

### 3.8 Clinical effectiveness of the nomogram model

**Figure 4:**
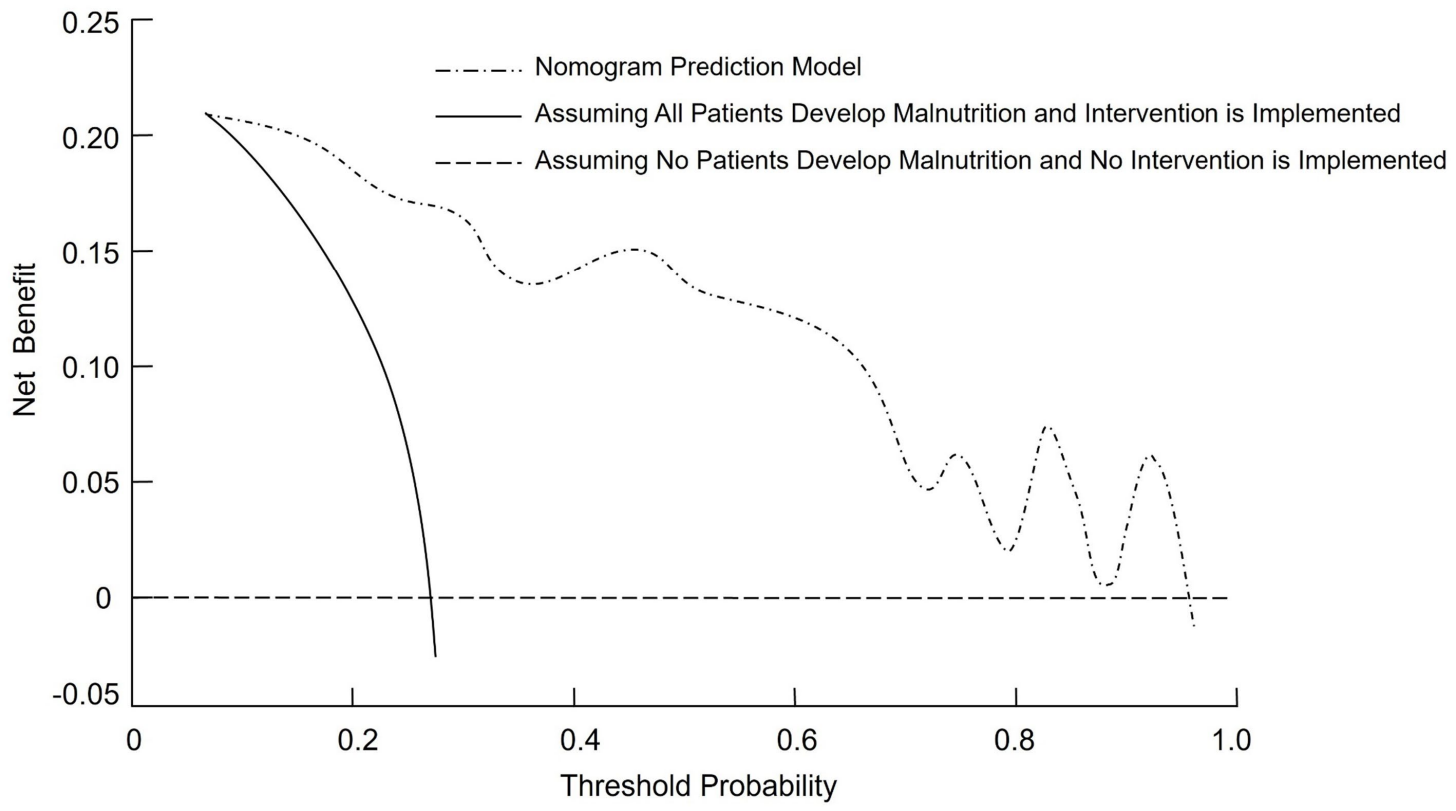
Decision curve analysis (DCA) curve for the nomogram model predicting malnutrition risk in CRC patients.

The DCA was performed to assess the clinical effectiveness of the nomogram model for predicting malnutrition in CRC patients. The straight line in the DCA represents the scenario where all patients are assumed to have malnutrition and receive intervention, resulting in a net benefit with a negative slope. The horizontal line reflects the situation where no patients are predicted to have malnutrition and no intervention is provided, leading to a net benefit of zero. The nomogram prediction model demonstrated a net benefit higher than both extreme scenarios across a wide range of threshold probabilities, suggesting potential clinical usefulness (Figure 4).

## 4 Discussion

Malnutrition is a common and serious complication in patients with CRC, significantly affecting treatment efficacy, recovery, and overall prognosis. It is associated with increased rates of postoperative complications, prolonged hospital stays, and diminished quality of life. Despite its high prevalence, accurately predicting malnutrition in CRC patients remains challenging due to the complex interplay of disease stage, treatment modalities, and individual patient characteristics. Early detection and timely nutritional intervention are essential for improving clinical outcomes and mitigating the adverse effects of malnutrition in this population (20). In the present study, we developed and validated a nomogram model for predicting malnutrition risk, incorporating key clinical variables such as age, tumor stage, duration of bed rest, KPS score, hemoglobin concentration, and serum PAB levels. The results underscore the multifactorial nature of malnutrition in CRC patients and highlight the necessity of a comprehensive, individualized approach to nutritional risk assessment and management.

Our findings demonstrated that patients aged 65 years or older were significantly more likely to be malnourished compared to younger individuals. This age-related disparity aligns with previous studies suggesting that aging is associated with an increased prevalence of malnutrition among cancer patients. Older adults often experience a combination of physiological changes, including diminished gastric motility, reduced appetite, and impaired nutrient absorption. In addition, the presence of chronic comorbidities such as diabetes and cardiovascular disease can further compromise nutritional status. The higher incidence of frailty and sarcopenia in this population exacerbates vulnerability to malnutrition, ultimately contributing to poorer clinical outcomes (21, 22). In our study, tumor stage emerged as a significant predictor of malnutrition. A markedly higher proportion of malnourished patients were classified as stage IV compared to those at earlier stages. This finding is consistent with existing literature demonstrating a strong association between advanced cancer stages and increased malnutrition risk. Disease progression in colorectal cancer often leads to elevated metabolic demands, systemic inflammation, and impaired nutrient utilization. Patients with advanced-stage CRC frequently experience gastrointestinal obstruction, persistent pain, and decreased oral intake, all of which contribute to nutritional decline. Moreover, anticancer treatments such as chemotherapy administered in later stages can further exacerbate nutritional deterioration, resulting in cancer-associated cachexia, a syndrome characterized by profound weight loss and skeletal muscle wasting (23, 24).

Prolonged bed rest and low KPS scores were also identified as significant risk factors for malnutrition in patients with colorectal cancer. Immobilization leads to skeletal muscle atrophy and reduced physical activity, which may further impair nutrient absorption and overall metabolic function. In addition, patients with low KPS scores, reflecting compromised functional capacity, often exhibit decreased appetite, increased fatigue, and limited mobility—factors that collectively contribute to nutritional deterioration. These observations highlight the importance of maintaining functional status and promoting mobility as part of comprehensive nutritional management strategies in CRC patients. Furthermore, our findings demonstrated strong associations between low HGB levels, reduced serum PAB concentrations, and malnutrition. Anemia, frequently observed in CRC patients due to chronic gastrointestinal blood loss, cytotoxic therapy, or insufficient nutrient intake, can significantly worsen the clinical burden of malnutrition. This relationship is reciprocal, as poor nutritional status can lead to deficiencies in essential hematopoietic micronutrients such as iron, folate, and vitamin B12. Low serum PAB, an established marker of visceral protein status, reflects inadequate protein intake or impaired protein metabolism—both indicative of malnourished states. These results underscore the need for routine monitoring of hematologic and nutritional biomarkers to enable early detection and timely intervention in patients at risk of malnutrition (25).

A nomogram is a graphical representation of a predictive model that integrates multiple variables to estimate an individual’s risk of a specific outcome, in this case malnutrition in CRC patients. The nomogram developed in this study offers a practical tool for clinicians to predict malnutrition risk in CRC patients. Incorporating key clinical variables such as age, TNM stage, functional status, and nutritional markers, the nomogram enables a comprehensive and individualized patient assessment. To facilitate its application, we provide the following example: consider a patient aged 70 years with TNM stage IV disease, a KPS score ≤80, prealbumin <200 g/L, hemoglobin <110 g/L, and prolonged bed rest. By locating each predictor on the nomogram and summing the assigned points, the total score can be mapped to a predicted probability of malnutrition. This visual tool enables rapid bedside estimation of risk without requiring complex computations. The nomogram showed acceptable predictive performance, with an AUC of 0.819, indicating moderate discriminative ability based on conventional ROC interpretation. This suggests the model can reasonably distinguish between malnourished and well-nourished patients. Regarding calibration, the model exhibited good agreement between predicted and observed probabilities, as evidenced by the calibration plot and the non-significant Hosmer–Lemeshow test (*p* = 0.929). These findings suggest consistent performance across the spectrum of risk, though we have limited our interpretation to reflect promising potential rather than conclusive clinical applicability. Additionally, DCA demonstrated that the model provided a net clinical benefit across a wide range of threshold probabilities compared to treating all or no patients. This supports its potential value in guiding clinical decision-making. Nonetheless, external validation in independent cohorts is necessary to confirm its generalizability and utility in routine practice. By stratifying patients based on their malnutrition risk, this model may facilitate timely, targeted nutritional interventions and help mitigate the adverse outcomes associated with malnutrition in CRC patients.

While this study provides valuable insights into the predictors of malnutrition in CRC patients, it is not without limitations. One important limitation is the lack of external validation which may limit its applicability to other populations or healthcare settings. Additionally, the retrospective nature of the analysis means that causality cannot be definitively established. Future studies should aim to validate the nomogram in larger, multi-center cohorts, and prospective studies are needed to confirm the effectiveness of the model in guiding clinical interventions. Furthermore, while we focused on traditional clinical and laboratory variables, other factors such as genetic predisposition, lifestyle habits, and the role of gut microbiota in CRC-related malnutrition remain underexplored. Incorporating these elements into future models could enhance their predictive accuracy and overall clinical utility.

## 5 Conclusion

In conclusion, advanced age, TNM stage IV, poor KPS scores, prolonged bed rest, decreased HGB, and lower PAB levels were identified as significant risk factors for malnutrition in colorectal cancer patients. The nomogram model developed based on these factors demonstrated good discrimination and predictive accuracy, offering valuable guidance for clinicians in preventing malnutrition in these patients.

## Data Availability

The raw data supporting the conclusions of this article will be made available by the authors, without undue reservation.
